# Survival Among Patients With High-Risk Gastrointestinal Cancers During the COVID-19 Pandemic

**DOI:** 10.1001/jamanetworkopen.2024.0160

**Published:** 2024-03-05

**Authors:** Lauren M. Janczewski, Amanda E. Browner, Joseph H. Cotler, Bryan E. Palis, Kelley Chan, Rachel H. Joung, David J. Bentrem, Ryan P. Merkow, Daniel J. Boffa, Heidi Nelson

**Affiliations:** 1 American College of Surgeons Cancer Programs, Chicago, Illinois; 2 Department of Surgery, Northwestern University Feinberg School of Medicine, Chicago, Illinois; 3 Department of Surgery, University of Chicago Pritzker School of Medicine, Chicago, Illinois; 4 Section of Thoracic Surgery, Department of Surgery, Yale School of Medicine, New Haven, Connecticut

## Abstract

**Importance:**

Prior reports demonstrated that patients with cancer experienced worse outcomes from pandemic-related stressors and COVID-19 infection. Patients with certain malignant neoplasms, such as high-risk gastrointestinal (HRGI) cancers, may have been particularly affected.

**Objective:**

To evaluate disruptions in care and outcomes among patients with HRGI cancers during the COVID-19 pandemic, assessing for signs of long-term changes in populations and survival.

**Design, Setting, and Participants:**

This retrospective cohort study used data from the National Cancer Database to identify patients with HRGI cancer (esophageal, gastric, primary liver, or pancreatic) diagnosed between January 1, 2018, and December 31, 2020. Data were analyzed between August 23 and September 4, 2023.

**Main Outcome and Measures:**

Trends in monthly new cases and proportions by stage in 2020 were compared with the prior 2 years. Kaplan-Meier curves and Cox regression were used to assess 1-year mortality in 2020 compared with 2018 to 2019. Proportional monthly trends and multivariable logistic regression were used to evaluate 30-day and 90-day mortality in 2020 compared with prior years.

**Results:**

Of the 156 937 patients included in this study, 54 994 (35.0%) were aged 60 to 69 years and 100 050 (63.8%) were men. There was a substantial decrease in newly diagnosed HRGI cancers in March to May 2020, which returned to prepandemic levels by July 2020. For stage, there was a proportional decrease in the diagnosis of stage I (−3.9%) and stage II (−2.3%) disease, with an increase in stage IV disease (7.1%) during the early months of the pandemic. Despite a slight decrease in 1-year survival rates in 2020 (50.7% in 2018 and 2019 vs 47.4% in 2020), survival curves remained unchanged between years (all *P* > .05). After adjusting for confounders, diagnosis in 2020 was not associated with increased 1-year mortality compared with 2018 to 2019 (hazard ratio, 0.99; 95% CI, 0.97-1.01). The rates of 30-day (2.1% in 2018, 2.0% in 2019, and 2.1% in 2020) and 90-day (4.3% in 2018, 4.4% in 2019, and 4.6% in 2020) operative mortality also remained similar.

**Conclusions and Relevance:**

In this retrospective cohort study, a period of underdiagnosis and increase in stage IV disease was observed for HRGI cancers during the pandemic; however, there was no change in 1-year survival or operative mortality. These results demonstrate the risks associated with gaps in care and the tremendous efforts of the cancer community to ensure quality care delivery during the pandemic. Future research should investigate long-term survival changes among all cancer types as additional follow-up data are accrued.

## Key Points

**Question:**

How did incidence, stage, and survival among patients with high-risk gastrointestinal (HRGI) cancers change after the start of the COVID-19 pandemic?

**Findings:**

In this cohort study with 156 937 patients, operative mortality and 1-year survival curves among patients with HRGI cancers were preserved during the pandemic. However, there was substantial underdiagnosis in 2020, with no proportional increase in newly diagnosed cases or stage migration throughout the remainder of the year.

**Meaning:**

These findings suggest that cancer clinicians continued to deliver quality care during 2020 for patients able to be diagnosed; however, underdiagnosis and lack of health care access may have also contributed to lives lost.

## Teaser

This cohort study uses data from the National Cancer Database to examine trends in incidence, stage, and survival among patients with high-risk gastrointestinal cancer during the COVID-19 pandemic.

## Introduction

The COVID-19 pandemic presented challenges to the delivery of cancer care.^1^ Resource diversion toward patients with COVID-19 led to a scarcity of personal protective equipment, health care personnel, and facility space that may have otherwise been used for patients with cancer.^2,3^ Thus, substantial decreases in cancer screening and diagnosis in 2020 have been described.^4,5^ Furthermore, previous work has shown the increased risk of worse outcomes for patients with cancer and COVID-19, especially in 2020, prior to the widespread availability of vaccinations. Specifically, early reports during the pandemic demonstrated that active COVID-19 infection was associated with increased perioperative and long-term mortality among patients with cancer.^6,7^

Although challenges in the delivery of care during the pandemic apply to all cancers, certain disease sites may have been particularly affected. For example, patients with high-risk gastrointestinal (HRGI) cancers, including esophageal, gastric, primary liver, and pancreatic cancers, already have higher rates of presenting at advanced stages as well as an increased risk of perioperative mortality and worse survival,^8,9,10,11,12^ which may have been exacerbated by COVID-19 infection or pandemic-related stressors. Thus, the frequency of newly diagnosed cases, staging, and mortality among patients with HRGI cancers during the pandemic may have been significantly altered and serves as a relevant case study for assessing changes in cancer care and outcomes during this time. However, the extent to which these critical cancer datapoints were affected among this population due to barriers created by the pandemic remains unknown.

The National Cancer Database (NCDB) identifies 72% of patients newly diagnosed with cancer in the US annually.^13^ Previous work analyzing the data collection infrastructure of the NCDB during the pandemic demonstrated that case abstraction remained intact, validating the use of this comprehensive, national database in future cohorts.^14^ Although prior studies have evaluated national-level changes in cancer diagnosis^5^ and treatment,^15,16,17,18^ additional site-specific analyses are essential to understand what happened as a result of the pandemic and to anticipate changes in patient needs moving forward. We hypothesized that during the onset of the pandemic, there was a period of underdiagnosis as well as an increase in patients presenting with advanced-stage disease. Additionally, we hypothesized that rates of 1-year and operative mortality increased during the pandemic, potentially due to pandemic-related stressors or COVID-19 infection. Thus, our objective was to evaluate the frequency of newly diagnosed cases, staging, and mortality, including overall 1-year survival and short-term operative mortality, among patients diagnosed with HRGI cancers during the COVID-19 pandemic.

## Methods

### Data Source

This retrospective cohort study queried the NCDB for patients diagnosed with HRGI cancer (esophageal, gastric, primary liver, or pancreatic) between January 1, 2018, and December 31, 2020, using data abstracted for the 2021 NCDB participant user file to ensure that all patients had a minimum of 12 months’ follow-up. The NCDB represents one of the most comprehensive cancer registries in the world^13,19^ and is jointly maintained by the American College of Surgeons Commission on Cancer and the American Cancer Society, abstracting data regarding the diagnosis and treatment of cancer at Commission on Cancer–accredited hospitals. All data within the NCDB are deidentified and Health Insurance Portability and Accountability Act compliant and thus deemed exempt from the American College of Surgeons institutional review board review. The study followed the Strengthening the Reporting of Observational Studies in Epidemiology (STROBE) reporting guideline.

### Patient Population

Patients aged 18 years or older with newly diagnosed esophageal, gastric, primary liver, or pancreatic cancer as well as patients with only 1 primary cancer diagnosis in their lifetime were included. These 4 disease sites were chosen specifically based on their propensity for worse survival outcomes and higher rates of operative mortality^10,11,12^ compared with other primary malignant neoplasms, representing a pertinent case study for detecting early changes to long-term outcomes during the pandemic. Data were defined using the *International Classification of Diseases for Oncology*, *Third Edition*, by primary site topography and histology codes.^20^ Patients with missing demographic or clinical staging data were excluded (eFigure 1 in Supplement 1).

### Independent Variables

The following variables were evaluated across the entire cohort: year of diagnosis, age at diagnosis (years), sex, race and ethnicity, facility type, insurance status, and Charlson-Deyo score (comorbidities).^21^ Importantly, month of diagnosis is not publicly available within the NCDB; however, because this analysis was performed by the American College of Surgeons Cancer Department staff, month of diagnosis was available and included. Race and ethnicity were self-reported and categorized as Asian including Hawaiian or Pacific Islander (hereinafter, Asian), Hispanic, non-Hispanic Black (hereinafter, Black), and non-Hispanic White (hereinafter, White). Racial and ethnic disparities in cancer care have been widely described before; thus, we adjusted for race and ethnicity in the current study. Among patients diagnosed with HRGI cancers in 2020, the proportion of patients diagnosed with COVID-19 was evaluated and stratified by perioperative infections, including 30 days before or after the date of definitive resection defined by prior studies.^6^

### Primary Outcomes

The primary outcomes were trends in newly diagnosed cases, stage at diagnosis, and mortality. Clinical stage was determined with the eighth edition of the American Joint Committee on Cancer staging system.^22^ Mortality included an evaluation of 1-year survival and operative mortality. The NCDB defines operative mortality as death within 30 or 90 days of the most definitive primary site surgery.^21^ Thus, evaluation of operative mortality was limited to patients who underwent curative-intent resection.

### Statistical Analysis

Patient demographic characteristics, facility type, disease site, and stage were evaluated across study years and compared using χ^2^ tests. To measure variance in the monthly reporting of HRGI cases, the number of newly diagnosed cases were plotted by month and compared across years using a repeated-measures analysis of variance (ANOVA) to assess for temporal changes. Next, the proportion of patients presenting at each stage was similarly plotted by month and compared across years with a repeated-measures ANOVA to measure changes in stage at diagnosis.

We then sought to evaluate potential changes in mortality during the pandemic, including 1-year overall survival and operative mortality rates. Kaplan-Meier methods in conjunction with log rank and Wilcoxon rank sum tests were used to assess 1-year survival between years. A multivariable Cox proportional hazards regression was used to assess the association of the year of diagnosis with survival, adjusting for patient demographic characteristics, facility type, disease site, and clinical stage and clustered by facility. To assess operative mortality, monthly trends in rates of both 30-day and 90-day mortality were compared across years using a repeated-measures ANOVA to identify potential increases in surgical risk during the pandemic among patients who underwent resection. Differences in operative mortality were additionally assessed between years and compared using χ^2^ tests. Multivariable logistic regression models, adjusting for patient demographics, facility type, disease site, and clinical stage, were used to evaluate the association of the diagnosis year with 30-day and 90-day operative mortality and similarly clustered by facility.

Recognizing that different primary disease sites may have been affected by pandemic-related stressors in different ways, sensitivity analyses were performed examining newly diagnosed cases, stage, and mortality across all 4 disease sites and studied as separate cohorts.

All statistical tests were 2-sided, with significance determined using a threshold of α < .05. All statistical analyses were conducted using SAS, version 9.4 (SAS Institute Inc). Data were analyzed between August 23 and September 4, 2023.

## Results

### Patient Characteristics

Overall, 156 937 patients with HRGI cancers were identified. Of these patients, 54 994 (35.0%) were aged between 60 and 69 years; 100 050 (63.8%) were men and 56 887 (36.2%) were women. In terms of race and ethnicity, 7969 (5.1%) of patients were Asian, 20 768 (13.2%) were Black, 15 437 (9.8%) were Hispanic, and 112 763 (71.9%) were White (Table). In total, 33.2% of patients were diagnosed in 2018, 34.5% in 2019, and 32.3% in 2020. Of the included patients, 17.4% had esophageal cancer, 13.5% had gastric cancer, 23.1% had primary liver cancer, and 45.9% had pancreatic cancer. Sociodemographic characteristics were similar among all years. In addition, 0.9% of patients diagnosed in 2020 had a positive COVID-19 test result at some point; of these, only 11.4% were in the perioperative period. Importantly, only 14.5% of patients in the study had documented COVID-19 test results.

**Table.** Characteristics of Patients Diagnosed With High-Risk Gastrointestinal Cancers, Years 2018-2020

Characteristic	Year of diagnosis, No. (%)			*P* value
	2018	2019	2020	
Age, y				
18-49	3581 (6.9)	3682 (6.8)	3424 (6.8)	.35
50-59	9926 (19.0)	9657 (17.9)	8663 (17.1)	
60-69	18 269 (35.0)	19 022 (35.2)	17 703 (34.9)	
70-79	13 563 (26.0)	14 373 (26.6)	14 094 (27.8)	
≥80	6824 (13.1)	7337 (13.6)	6819 (13.4)	
Sex				
Male	33 441 (64.1)	34 390 (63.6)	32 219 (63.5)	.11
Female	18 722 (35.9)	19 681 (36.4)	18 484 (36.5)	
Race and ethnicity				
Asian including Hawaiian or Pacific Islander	2626 (5.0)	2776 (5.1)	2567 (5.1)	.20
Hispanic	5078 (9.7)	5419 (10.0)	4940 (9.7)	
Non-Hispanic Black	7045 (13.5)	7090 (13.1)	6633 (13.1)	
Non-Hispanic White	37 414 (71.7)	38 786 (71.7)	36 563 (72.1)	
Facility type				
Academic	24 776 (47.5)	25 242 (46.7)	22 398 (44.2)	<.001
Community	1887 (3.6)	1851 (3.4)	1978 (3.9)	
Comprehensive community	13 837 (26.5)	14 662 (27.1)	14 493 (28.6)	
Integrated	11 663 (22.4)	12 316 (22.8)	11 834 (23.3)	
Insurance type				
Private	15 713 (30.1)	15 831 (29.3)	14 652 (28.9)	<.001
Medicaid	5370 (10.3)	5441 (10.1)	5057 (10.0)	
Medicare	28 293 (54.2)	29 846 (55.2)	28 274 (55.8)	
Not insured	1726 (3.3)	1815 (3.4)	1549 (3.1)	
Other government	1061 (2.0)	1138 (2.1)	1171 (2.3)	
Charlson-Deyo score				
0	31 488 (60.4)	32 703 (60.5)	30 651 (60.5)	.88
1	10 766 (20.6)	11 142 (20.6)	10 326 (20.4)	
2	4417 (8.5)	4569 (8.5)	4315 (8.5)	
≥3	5492 (10.5)	5657 (10.4)	5411 (10.6)	
Disease site				
Esophagus	9105 (17.5)	9479 (17.5)	8794 (17.3)	<.001
Gastric	7192 (13.8)	7410 (13.7)	6630 (13.1)	
Liver	12 337 (23.7)	12 686 (23.5)	11 214 (22.1)	
Pancreas	23 529 (45.1)	24 496 (45.3)	24 065 (47.5)	
Clinical stage^a^				
I	12 378 (23.7)	13 082 (24.2)	11 916 (23.5)	<.001
II	8384 (16.1)	8552 (15.8)	7658 (15.1)	
III	9548 (18.3)	9599 (17.8)	8874 (17.5)	
IV	21 853 (41.9)	22 838 (42.2)	22 255 (43.9)	

^a^ Clinical stage of disease was determined using the eighth edition of the American Joint Committee on Cancer staging system.^22^

### Frequency of Newly Diagnosed HRGI Cancers

**Figure 1.**
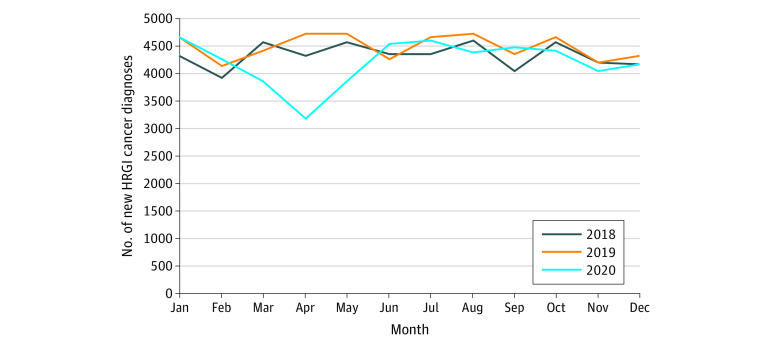
Monthly Incidence of High-Risk Gastrointestinal (HRGI) Cancer Diagnoses in 2018 to 2020 *P* = .002 (repeated-measures analysis of variance).

In general, there was a significant decrease in newly diagnosed HRGI cancers in March through May 2020 compared with prior years, representing at least 3000 fewer cases during this time and greater than 1500 fewer new cases in April 2020 alone (*P* = .002; Figure 1). However, the frequency of newly diagnosed cases per month returned to prepandemic levels by July 2020, with no evidence of a proportional increase, or rebound throughout the remainder of 2020.

### Stage at Diagnosis

**Figure 2.**
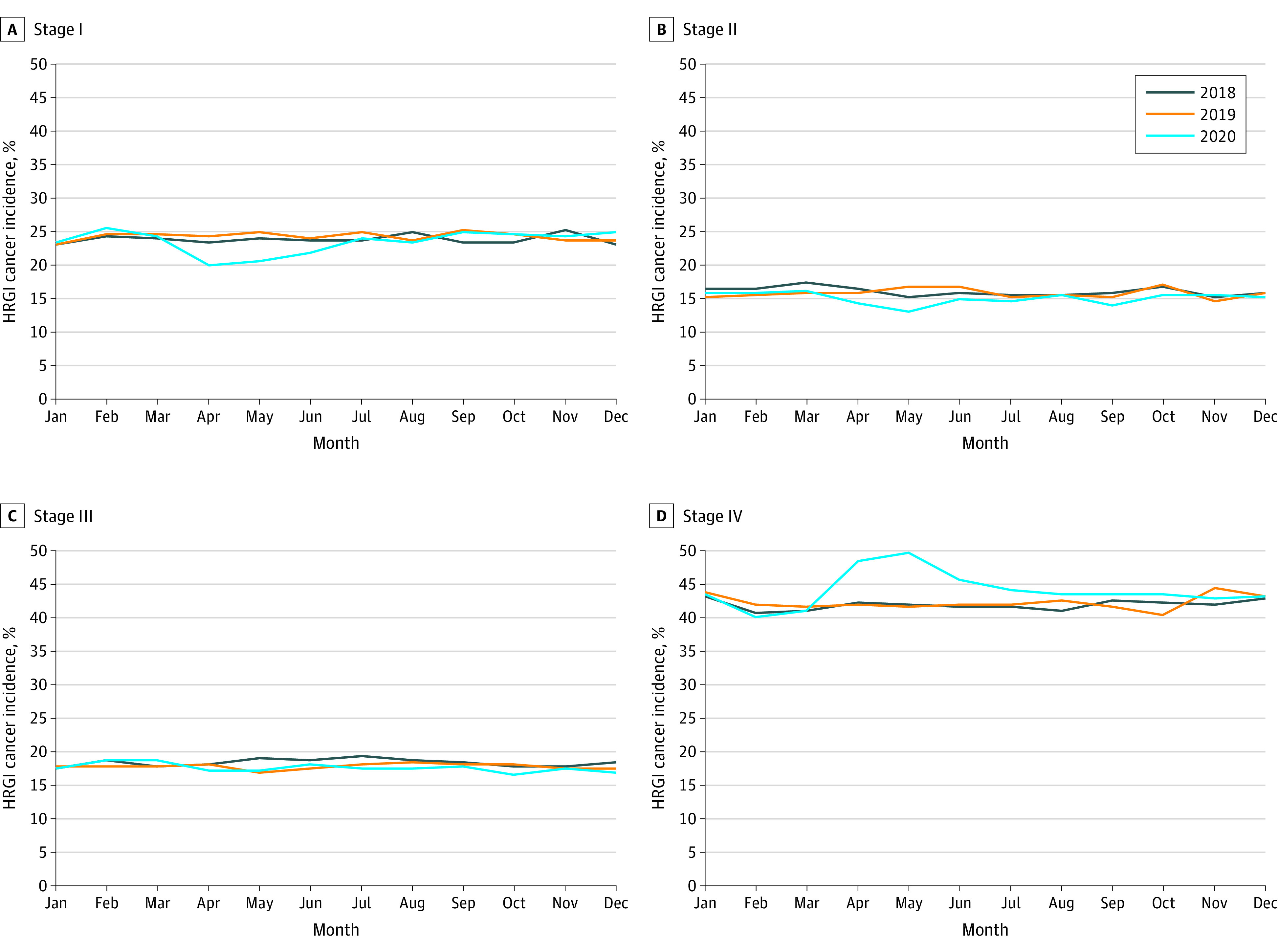
Monthly Incidence of High-Risk Gastrointestinal (HRGI) Cancers by Stage in 2018 to 2020 Results are presented for stages I (A), II (B), III (C), and IV (D). *P* < .001 (repeated-measures analysis of variance).

There was a slight increase in the proportion of patients presenting with stage IV disease in 2020 (43.9%) compared with 2018 (41.9%) and 2019 (42.2%) (*P* < .001; Table). When trends throughout the year were evaluated, there was a noticeable decrease in patients diagnosed with stage I (−3.9%) and stage II (−2.3%) disease as well as a dramatic increase in patients presenting with stage IV disease (7.1%), primarily in March through May 2020 (*P* < .001; Figure 2). However, throughout the remainder of 2020, the proportion of patients presenting at each stage mirrored the trends in stage observed in prior years.

### Mortality

**Figure 3.**
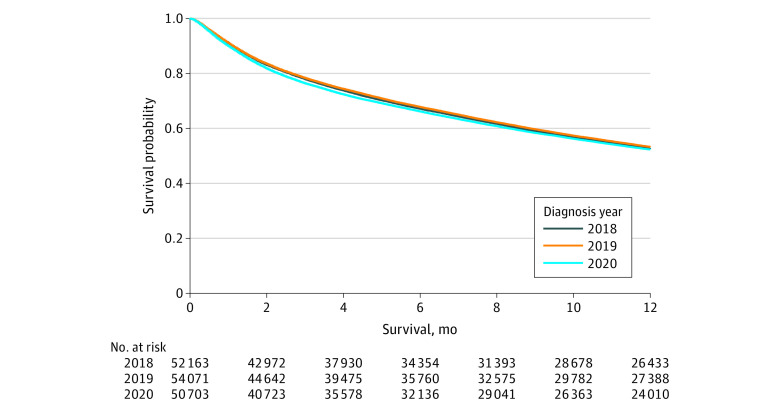
One-Year Survival Among Patients With High-Risk Gastrointestinal (HRGI) Cancers Diagnosed in 2018 to 2020 *P* = .30 (log rank test) and *P* = .20 (Wilcoxon rank sum test).

When mortality was evaluated, 1-year survival rates in 2020 were 47.4% compared with 50.7% in both 2018 and 2019 (*P* < .001; eTable 1 in Supplement 1). Despite this variation, 1-year survival curves for 2020 reflected those of 2018 and 2019 (log rank *P* = .30, Wilcoxon *P* = .20; Figure 3). These findings were similarly demonstrated in a multivariable Cox regression, in which patients diagnosed in 2020 were not more likely to experience mortality at 1 year (hazard ratio, 0.99; 95% CI, 0.97-1.01) compared with prior years after adjusting for potential confounders. Importantly, after repeating this analysis with only stages I through III disease, 1-year survival rates in 2020 were 64.1% compared with 67.9% in 2018 and 67.7% in 2019 (*P* < .001). Despite a similar variation as within the overall cohort, our findings remained such that patients diagnosed in 2020 were not more likely to experience 1-year mortality on a multivariable Cox regression (hazard ratio, 0.98; 95% CI, 0.96-1.03).

**Figure 4.**
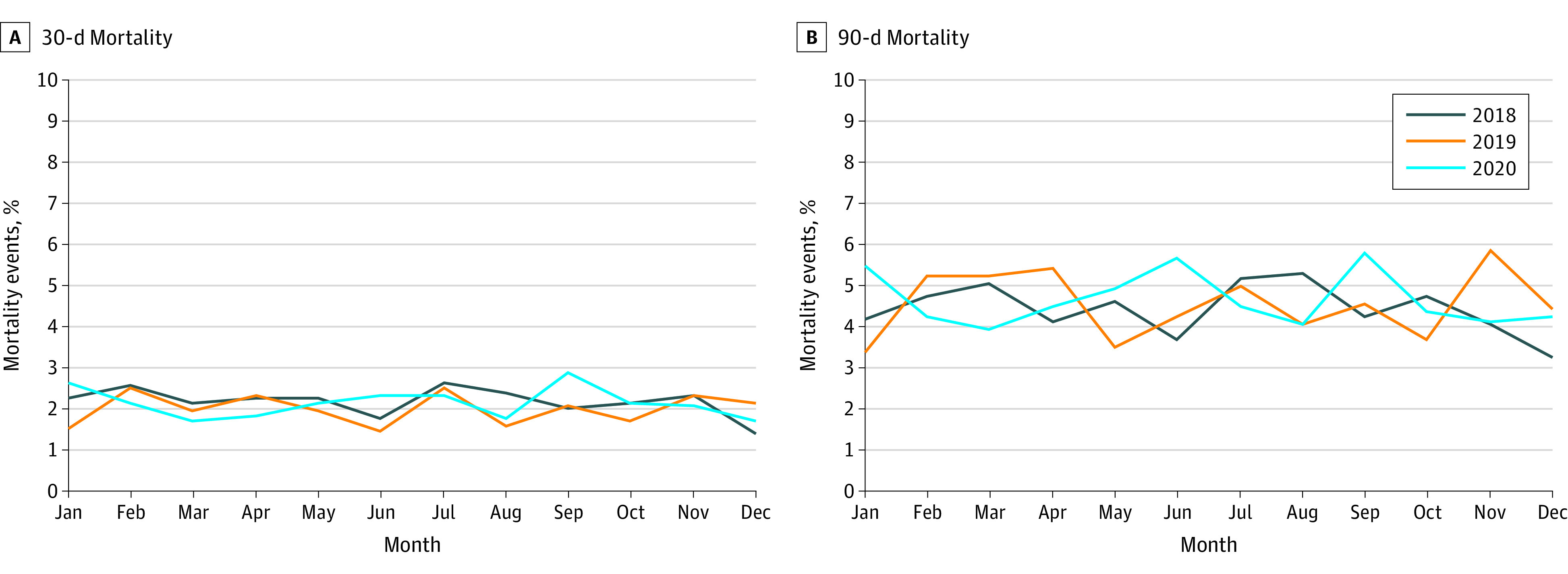
Mortality Events by Month Among Patients With High-Risk Gastrointestinal (HRGI) Cancers in 2018 to 2020 Percentage of mortality events at 30 days (*P* = .04) (A) and 90 days (*P* = .10) (B) (repeated-measures analysis of variance).

For operative mortality, 39 412 patients underwent definitive resection. Of these patients, there was a slight decrease in the proportion of patients undergoing surgery in 2020 (25.7% in 2018, 25.3% in 2019, and 24.3% in 2020; *P* < .001; eTable 2 in Supplement 1). Similarly, changes in the proportion of patients treated with chemotherapy and radiation were small between years. Importantly, although there was slight variance in the monthly rate of 30-day mortality events (2.1% in 2018, 2.0% in 2019, and 2.1% in 2020; *P* = .04; Figure 4A), the plotted trends did not reflect worse outcomes during the early months of the pandemic, and no variance in 90-day mortality was observed (4.3% in 2018, 4.4% in 2019, and 4.6% in 2020; *P* = .10; Figure 4B). Although there was a small increase in the proportion of unknown operative mortality data in 2020 (from 0.6% to 2.1%; *P* < .001), changes in the rate of operative mortality between years were minimal (eTable 1 in Supplement 1). These results remained consistent on multivariable analysis as well; after adjusting for potential confounders, patients were not more likely to experience 30-day (odds ratio, 0.96; 95% CI 0.81-1.14) or 90-day (odds ratio, 1.04; 95% CI 0.92-1.17) operative mortality in 2020 compared with prior years.

### Sensitivity Analysis

Recognizing the potential for site-specific differences, trends in newly diagnosed cases, staging, and mortality were repeated across the 4 cancer sites included in this analysis. Specifically, similar trends in case counts were seen across all 4 diseases, with the most substantial decreases exhibited for primary liver and pancreatic cancers (eFigure 2 in Supplement 1). We similarly identified a slight increase in the proportion of patients presenting with stage IV disease, most noticeably for gastric cancer (eTable 3 in Supplement 1). In addition, although there was a slight decrease in 1-year survival in 2020, rates of operative mortality reflected those of the larger cohort across years (eTable 4 in Supplement 1), with 1-year survival curves mirroring those of prior years as well (eFigure 3 in Supplement 1).

## Discussion

The delivery of cancer care was undoubtedly affected during the COVID-19 pandemic.^4,5,23^ Early reports revealing increased postoperative mortality due to COVID-19 infection^6^ influenced the creation of consensus statements regarding the triage of elective procedures, often including cancer operations.^24,25^ These guidelines may have particularly affected certain populations, such as those with HRGI cancers, in which resection is the only chance for cure and delays in management may lead to worse outcomes. The findings of this national study of patients with newly diagnosed HRGI cancers suggest substantial underdiagnosis in the first year of the pandemic. Additionally, patients more frequently presented with advanced-stage disease; however, at a time when the pandemic was disrupting health care, it is a tribute to the efforts of cancer clinicians that 1-year survival curves and operative mortality remained unchanged, as our findings suggest.

There was a considerable decrease in newly diagnosed HRGI cancers at the onset of the pandemic.^5^ However, as 2020 progressed, the frequency of newly diagnosed cases returned to prepandemic levels, which is consistent with previous reports.^5^ Obviously, there was not a decrease in the number of patients who developed cancer as a result of the pandemic; rather, notably fewer patients were diagnosed, likely due to pandemic-related stressors. To the credit of the cancer programs, the findings of this study suggest that the frequency of newly diagnosed HRGI cancers quickly recovered within the second half of 2020. Because more than 3000 fewer cases were reported at the beginning of the pandemic, it is likely that the early lack of available health care, including scarce resources and fears of contracting COVID-19, led to a reluctance to seek medical care^2,3,26,27^ and may have resulted in substantial loss of life for patients with HRGI cancers who would have otherwise had access to diagnosis and treatment. Specifically, these data call attention to a critical health care systems failure in our ability to respond to national crises. Although the COVID-19 public health emergency has been lifted, these results are broadly applicable to future pandemics, national emergencies, and natural disasters, demonstrating the need for improved safeholds to ensure future lives are not lost unnecessarily.

We also observed a substantial increase in the diagnosis of advanced-stage disease at the onset of the pandemic. These findings are consistent with prior reports across numerous other cancer sites^5^ and, thus, are more likely representative of the time during the pandemic when the patient sought care rather than true evidence of stage migration. Specifically, during the early months of the pandemic, patients with milder symptoms may have delayed seeking medical advice, whereas those with severe symptoms due to advanced disease had to seek care by necessity. This represents an opportunity to improve patient education by advising them to seek medical attention when any symptoms arise rather than just severe ones. In addition, given that screening mechanisms are absent for detecting HRGI cancers, timely diagnosis is dependent on primary care physician referrals, usually after the development of early-onset symptoms, followed by imaging, laboratory tests, or endoscopy. However, delays in routine primary care health checks, along with office closures and cancellations of elective procedures such as endoscopy, may have contributed to the proportional decrease in the diagnosis of early-stage disease.^28,29^ Furthermore, to evaluate the association of the pandemic with other aspects of cancer care such as screening, future work should evaluate changes in the presentation of screenable cancers as well as their long-term outcomes.

Despite the observed shifts in stage, we identified no difference in 1-year survival curves for patients diagnosed with HRGI cancers in 2020. These findings are contradictory to the hypotheses described in numerous prior studies,^5,30^ illustrating diagnostic and treatment delays that were projected to substantially affect survivorship.^31,32^ However, prior work using the NCDB to evaluate alterations in cancer treatment during the pandemic demonstrated a substantial decrease in time to treatment initiation^18^ which may contribute to the consistency in survival curves in the current study. Although this cohort of patients will be followed over time, it is unlikely that mortality rates at further time points will differ substantially, given the present results. These data highlight a key point for cancer researchers across the US, that inclusion of this cohort in future studies is unlikely to confound survival analyses moving forward.

In this study, operative mortality rates remained stable compared with prior years, without evidence of deviation throughout the first year of the pandemic. These data highlight the tremendous efforts of cancer clinicians at the time, who came together on behalf of patients with cancer to ensure that they continued receiving quality care despite being faced with one of the deadliest pandemics in history.^33,34^ This is consistent with prior studies that showed success in continuing to perform necessary cancer operations to prevent lapses in treatment. For example, a COVID-minimal surgical pathway was created to standardize best practices in preventing COVID-19 contraction among surgical patients, demonstrating that procedures could still safely be performed.^35^ Although highly impactful literature identified that infection with COVID-19 was associated with increased operative mortality^6^ and led to the development of guidelines on the appropriate triage of patients requiring surgery,^24,25,36^ patients with HRGI cancers still safely received cancer care.

### Limitations

This study has several limitations. First, although prior work demonstrated that the data collection infrastructure of the NCDB during the pandemic remained intact,^14^ this work evaluated the NCDB in its entirety and site-specific changes, such as for HRGI cancers, may exist. Although there was a statistically significant increase in the proportion of missing operative mortality data in 2020, the absolute difference in the number and frequency of these missing data compared with prior years was slight and likely of little clinical significance. Second, a small proportion of patients in this study were diagnosed with COVID-19; this finding is limited by only 14.5% of patients having a documented test result, preventing our evaluation of outcomes among patients diagnosed with COVID-19 in the perioperative period. Although the time to widely available COVID-19 testing likely contributed to this finding, it is also possible that mortality rates were preserved in this study because patients with undiagnosed cancer may have died from COVID-19 prior to having the opportunity to undergo surgery or other cancer treatment. Third, consequences of the pandemic were felt differently throughout the US at different times because it started at the coasts and progressed inward.^37^ Thus, these results may not be reflective of the experience at any one place or time during 2020.

## Conclusions

The findings of this cohort study suggest that the frequency of newly diagnosed HRGI cancers substantially decreased overall during the height of the COVID-19 pandemic, leaving more than 3000 fewer patients diagnosed in 2020. Among patients diagnosed with HRGI cancers in 2020, both 1-year survival curves and operative mortality rates remained stable despite an increase in patients presenting with stage IV disease. These results highlight the countervailing risks of health care disruption and remarkable work of the cancer community to continue providing quality cancer care during the first year of the pandemic. Future research should investigate long-term survival changes among all cancer types as additional follow-up data are accrued.

## Notes

Supplement 1. **eFigure 1.** CONSORT Diagram **eTable 1.** Overall 1-Year Mortality, 30-Day Operative Mortality, and 90-Day Operative Mortality by Year Among Patients With High-Risk Gastrointestinal Cancers in 2018 to 2020 **eTable 2.** Treatment of High-Risk Gastrointestinal Cancer Diagnoses in 2018 to 2020 **eFigure 2.** Monthly Incidence of High-Risk Gastrointestinal Cancer Diagnoses in 2018 to 2020 for Esophageal, Gastric, Primary Liver, and Pancreatic Cancers **eTable 3.** Clinical Stage at Diagnosis by Year Among Patients With Esophageal, Gastric, Primary Liver, and Pancreatic Cancers Diagnosed in 2018 to 2020 **eTable 4.** Overall 1-Year Mortality, 30-Day Operative Mortality, and 90-Day Operative Mortality by Year Among Patients With Esophageal, Gastric, Primary Liver, and Pancreatic Cancers Diagnosed in 2018 to 2020 **eFigure 3.** One-Year Survival Among Patients With Esophageal, Gastric, Primary Liver, and Pancreatic Cancers Diagnosed in 2018 to 2020

Supplement 2.
Data Sharing Statement
