# Supplementary material for: Survival Among Patients With High-Risk Gastrointestinal Cancers During the COVID-19 Pandemic
Source: JAMA Netw Open. 2024 Mar 5;7(3):e240160. doi: 10.1001/jamanetworkopen.2024.0160 (PMC10915687; doi:10.1001/jamanetworkopen.2024.0160)
Supplement: Supplement 1. — eFigure 1. CONSORT Diagram eTable 1. Overall 1-Year Mortality, 30-Day Operative Mortality, and 90-Day Operative Mortality by Year Among Patients With High-Risk Gastrointestinal Cancers in 2018 to 2020 eTable 2. Treatment of High-Risk Gastrointestinal Cancer Diagnoses in 2018 to 2020 eFigure 2. Monthly Incidence of High-Risk Gastrointestinal Cancer Diagnoses in 2018 to 2020 for Esophageal, Gastric, Primary Liver, and Pancreatic Cancers eTable 3. Clinical Stage at Diagnosis by Year Among Patients With Esophageal, Gastric, Primary Liver, and Pancreatic Cancers Diagnosed in 2018 to 2020 eTable 4. Overall 1-Year Mortality, 30-Day Operative Mortality, and 90-Day Operative Mortality by Year Among Patients With Esophageal, Gastric, Primary Liver, and Pancreatic Cancers Diagnosed in 2018 to 2020 eFigure 3. One-Year Survival Among Patients With Esophageal, Gastric, Primary Liver, and Pancreatic Cancers Diagnosed in 2018 to 2020 [file jamanetwopen-e240160-s001.pdf]

Supplementary Online Content

Janczewski LM, Browner AE, Cotler JH, et al. Survival among patients with high-risk gastrointestinal cancers during the COVID-19 pandemic. *JAMA Netw Open*. 2024;7(3):e240160. doi:10.1001/jamanetworkopen.2024.0160

eFigure 1. CONSORT Diagram

eTable 1. Overall 1-Year Mortality, 30-Day Operative Mortality, and 90-Day Operative Mortality by Year Among Patients With High-Risk Gastrointestinal Cancers in 2018 to 2020

eTable 2. Treatment of High-Risk Gastrointestinal Cancer Diagnoses in 2018 to 2020

eFigure 2. Monthly Incidence of High-Risk Gastrointestinal Cancer Diagnoses in 2018 to 2020 for Esophageal, Gastric, Primary Liver, and Pancreatic Cancers

eTable 3. Clinical Stage at Diagnosis by Year Among Patients With Esophageal, Gastric, Primary Liver, and Pancreatic Cancers Diagnosed in 2018 to 2020

eTable 4. Overall 1-Year Mortality, 30-Day Operative Mortality, and 90-Day Operative Mortality by Year Among Patients With Esophageal, Gastric, Primary Liver, and Pancreatic Cancers Diagnosed in 2018 to 2020

eFigure 3. One-Year Survival Among Patients With Esophageal, Gastric, Primary Liver, and Pancreatic Cancers Diagnosed in 2018 to 2020

This supplementary material has been provided by the authors to give readers additional information about their work.

© 2024 Janczewski LM et al. *JAMA Network Open.*

eFigure 1. CONSORT Diagram

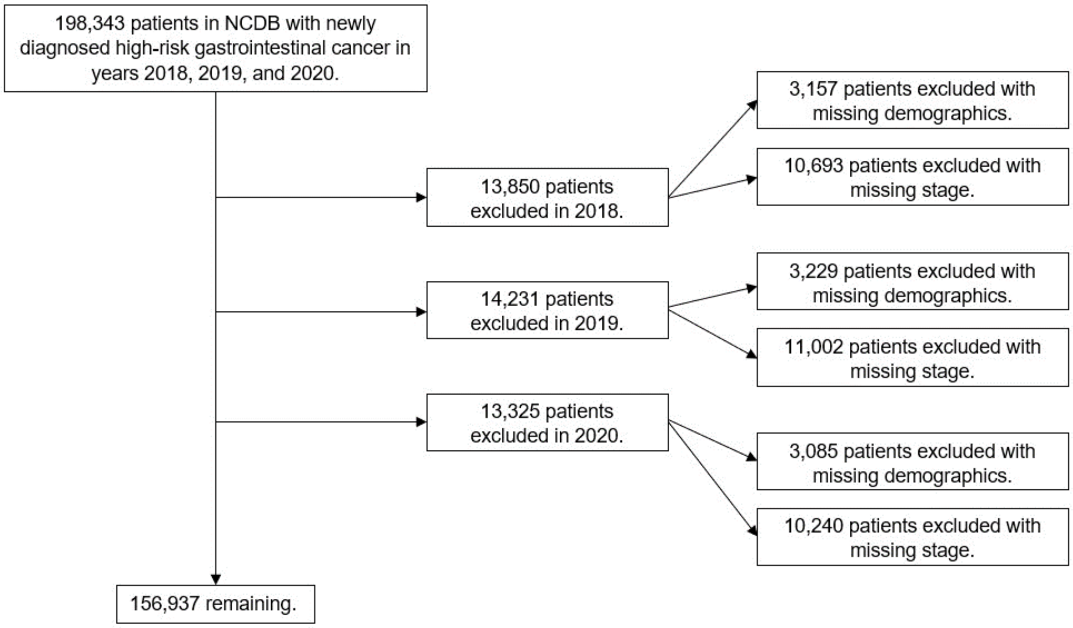

© 2024 Janczewski LM et al. *JAMA Network Open.*

eTable 1. Overall 1-Year Mortality, 30-Day Operative Mortality, and 90-Day Operative Mortality by Year Among Patients With High-Risk Gastrointestinal Cancers in 2018 to 2020

	Year			
	**2018, N (%)**	**2019, N (%)**	**2020, N (%)**	**p-value**
Alive at 1-year[a]	26,458 (50.7)	27,396 (50.7)	24,023 (47.4)	<0.001
1-year Mortality	25,705 (49.3)	26,675 (49.3)	26,680 (52.6)	
Alive at 30-days[b]	13,070 (97.4)	13,319 (97.5)	11,908 (96.5)	<0.001
30-day Mortality	282 (2.1)	269 (2.0)	260 (2.1)	
Unknown	64 (0.5)	69 (0.5)	171 (1.4)	
Alive at 90-days[b]	12,730 (94.9)	12,897 (94.5)	11,429 (92.6)	<0.001
90-day Mortality	581 (4.3)	607 (4.4)	568 (4.6)	
Unknown	105 (0.8)	153 (1.1)	342 (2.8)	

[a] Includes the entire patient cohort of 156,937 patients diagnosed with high-risk gastrointestinal cancers (52,163 in 2018, 54,071 in 2019, and 50,703 in 2020).

[b] Represents short-term operative mortality only and thus limited to only patients who underwent curative-intent surgical resection, 39,412 patients (13,416 in 2018, 13,657 in 2019, and 12,339 in 2020).

© 2024 Janczewski LM et al. *JAMA Network Open.*

eTable 2. Treatment of High-Risk Gastrointestinal Cancer Diagnoses in 2018 to 2020

	Year			p-value
	**2018, N (%)**	**2019, N (%)**	**2020, N (%)**	
Surgery	13,416 (25.7)	13,657 (25.3)	12,339 (24.3)	<0.001
No Surgery	36,782 (70.5)	38,363 (70.9)	36,583 (71.2)	
Unknown	1,965 (3.8)	2,051 (3.8)	1,781 (3.5)	
Systemic Therapy	28,892 (55.4)	29,903 (55.3)	27,359 (54.0)	<0.001
No Systemic Therapy	22,719 (43.6)	23,612 (43.7)	22,842 (45.1)	
Unknown	552 (1.1)	556 (1.0)	502 (0.9)	
Radiotherapy	13,435 (25.8)	13,875 (25.7)	12,552 (24.8)	<0.001
No Radiotherapy	38,496 (73.9)	39,932 (73.8)	37,873 (74.7)	
Unknown	232 (0.4)	264 (0.5)	278 (0.5)	

© 2024 Janczewski LM et al. *JAMA Network Open.*

eFigure 2. Monthly Incidence of High-Risk Gastrointestinal Cancer Diagnoses in 2018 to 2020 for Esophageal, Gastric, Primary Liver, and Pancreatic Cancers

a) Esophageal cancer

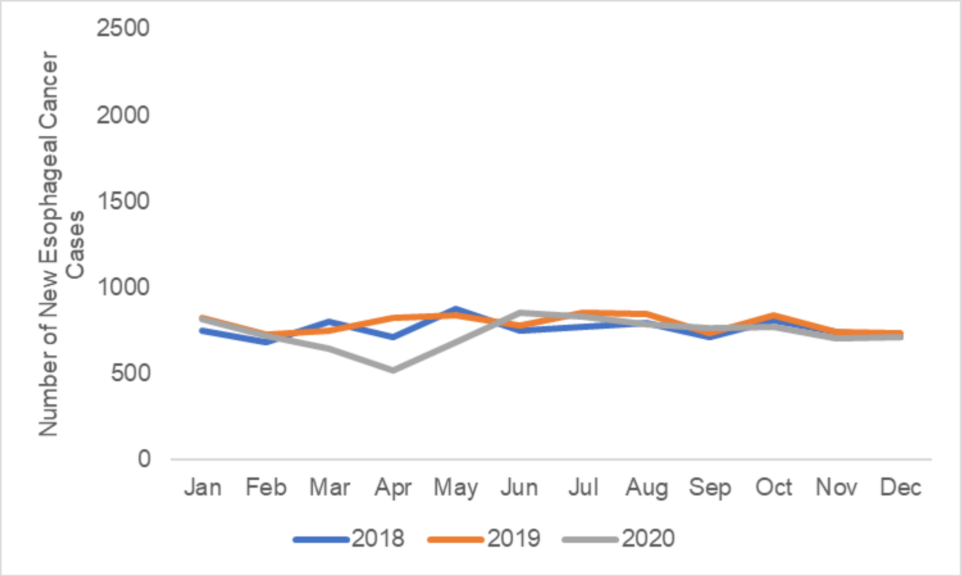

b) Gastric cancer

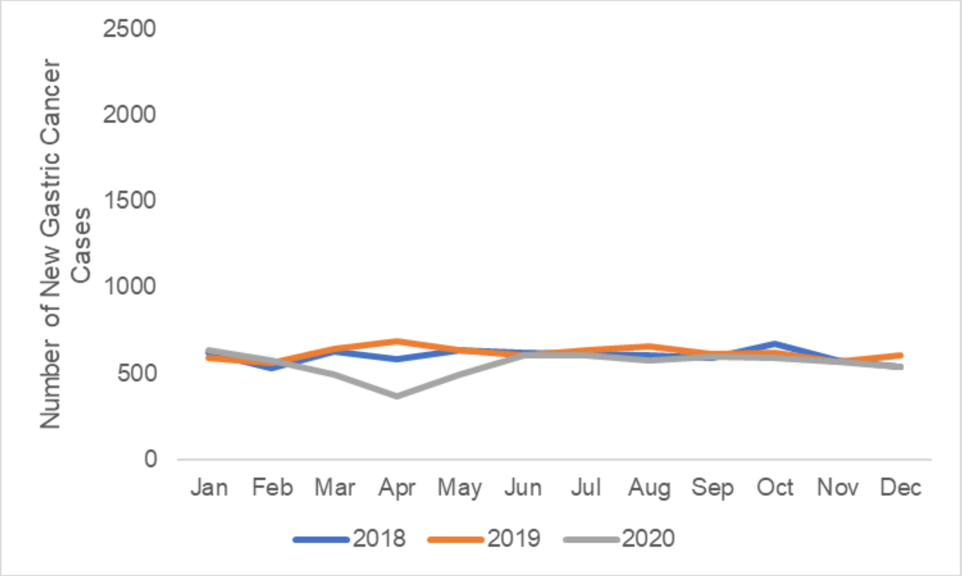

© 2024 Janczewski LM et al. *JAMA Network Open.*

c) Primary liver cancer

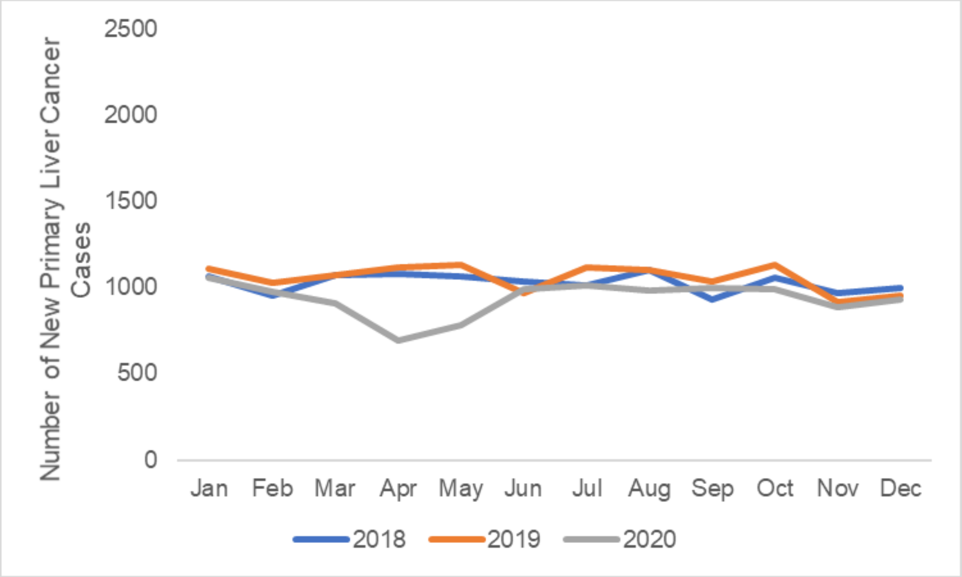

d) Pancreatic cancer

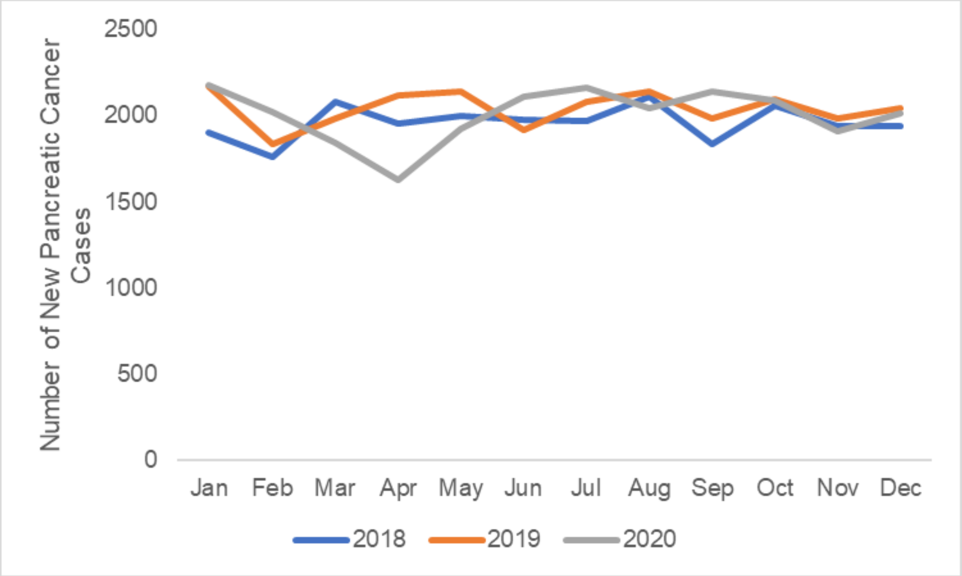

© 2024 Janczewski LM et al. *JAMA Network Open.*

eTable 3. Clinical Stage at Diagnosis by Year Among Patients With Esophageal, Gastric, Primary Liver, and Pancreatic Cancers Diagnosed in 2018 to 2020

		Year		
		**2018, N (%)**	**2019, N (%)**	**2020, N (%)**
**Esophagus**	*Stage I*	917 (10.1)	944 (10.0)	791 (9.0)
	Stage II	1,063 (11.7)	1,068 (11.3)	974 (11.1)
	Stage III	2,985 (32.8)	3,001 (31.7)	2,737 (31.1)
	Stage IV	4,140 (45.5)	4,466 (47.1)	4,292 (48.8)
**Gastric**	*Stage I*	1,697 (23.6)	1,796 (24.2)	1,466 (22.1)
	Stage II	1,060 (14.7)	1,102 (14.9)	887 (13.4)
	Stage III	970 (13.5)	988 (13.3)	881 (13.3)
	Stage IV	3,465 (48.2)	3,524 (47.6)	3,396 (51.2)
**Liver**	*Stage I*	4,358 (35.3)	4,406 (34.7)	3,786 (33.8)
	Stage II	2,759 (22.4)	2,842 (22.4)	2,350 (21.0)
	Stage III	2,850 (23.1)	2,916 (23.0)	2,617 (23.3)
	Stage IV	2,370 (19.2)	2,522 (19.9)	2,461 (21.9)
**Pancreas**	*Stage I*	5,406 (23.0)	5,936 (24.2)	5,873 (24.4)
	Stage II	3,502 (14.9)	3,540 (14.5)	3,447 (14.3)
	Stage III	2,743 (11.7)	2,694 (11.0)	2,639 (11.0)
	Stage IV	11,878 (50.5)	12,326 (50.3)	12,106 (50.3)

© 2024 Janczewski LM et al. *JAMA Network Open.*

eTable 4. Overall 1-Year Mortality, 30-Day Operative Mortality, and 90-Day Operative Mortality by Year Among Patients With Esophageal, Gastric, Primary Liver, and Pancreatic Cancers Diagnosed in 2018 to 2020

		Year		
		**2018, N (%)**	**2019, N (%)**	**2020, N (%)**
**Esophagus**	*Alive at 1-year*[a]	5,371 (59.0)	5,464 (57.6)	4,870 (55.4)
	1-year Mortality	3,734 (41.0)	4,015 (42.4)	3,924 (44.6)
	Alive at 30-days[b]	3,219 (97.1)	3,214 (97.5)	2,794 (96.3)
	30-day Mortality	81 (2.4)	69 (2.1)	84 (2.9)
	Unknown	15 (0.5)	13 (0.4)	23 (0.8)
	Alive at 90-days[b]	3,109 (93.8)	3,078 (93.4)	2,665 (91.9)
	90-day Mortality	186 (5.6)	191 (5.8)	181 (6.2)
	Unknown	20 (0.6)	27 (0.8)	55 (1.9)
**Gastric**	*Alive at 1-year*[a]	4,253 (59.1)	4,329 (58.4)	3,574 (53.9)
	1-year Mortality	2,939 (40.9)	3,081 (41.6)	3,056 (46.1)
	Alive at 30-days[b]	2,996 (97.7)	3,008 (97.4)	2,451 (96.2)
	30-day Mortality	50 (1.6)	49 (1.6)	45 (1.8)
	Unknown	21 (0.7)	30 (1.0)	52 (2.0)
	Alive at 90-days[b]	2,923 (95.3)	2,932 (95.0)	2,353 (92.3)
	90-day Mortality	106 (3.5)	98 (3.2)	104 (4.1)
	Unknown	38 (1.2)	57 (1.9)	91 (3.6)
**Liver**	*Alive at 1-year*[a]	6,946 (56.3)	7,139 (56.3)	5,822 (51.9)
	1-year Mortality	5,391 (43.7)	5,547 (43.7)	5,392 (48.1)
	Alive at 30-days[b]	1,501 (96.6)	1,541 (97.0)	1,394 (97.1)
	30-day Mortality	47 (3.0)	44 (2.8)	18 (1.3)
	Unknown	6 (0.4)	4 (0.3)	23 (1.6)
	Alive at 90-days[b]	1,478 (95.1)	1,508 (94.9)	1,337 (93.2)
	90-day Mortality	68 (4.4)	73 (4.6)	47 (3.3)
	Unknown	8 (0.5)	8 (0.5)	51 (3.5)
**Pancreas**	*Alive at 1-year*[a]	9,888 (42.0)	10,464 (42.7)	9,757 (40.5)
	1-year Mortality	13,641 (58.0)	14,032 (57.3)	14,308 (59.5)
	Alive at 30-days[b]	5,354 (97.7)	5,556 (97.7)	5,269 (96.6)
	30-day Mortality	104 (1.9)	107 (1.9)	113 (2.1)
	Unknown	22 (0.4)	22 (0.4)	73 (1.3)

© 2024 Janczewski LM et al. *JAMA Network Open.*

	Alive at 90-days[b]	5,220 (95.3)	5,379 (94.6)	5,074 (93.0)
	90-day Mortality	221 (4.0)	245 (4.3)	236 (4.3)
	Unknown	39 (0.7)	61 (1.1)	145 (2.7)

[a] Includes the entire patient cohort of 156,937 patients diagnosed with high-risk gastrointestinal cancers (52,163 in 2018, 54,071 in 2019, and 50,703 in 2020).

[b] Represents short-term operative mortality only and thus limited to only patients who underwent curative-intent surgical resection, 39,412 patients (13,416 in 2018, 13,657 in 2019, and 12,339 in 2020).

© 2024 Janczewski LM et al. *JAMA Network Open.*

eFigure 3. One-Year Survival Among Patients With Esophageal, Gastric, Primary Liver, and Pancreatic Cancers Diagnosed in 2018 to 2020

a) Esophageal cancer

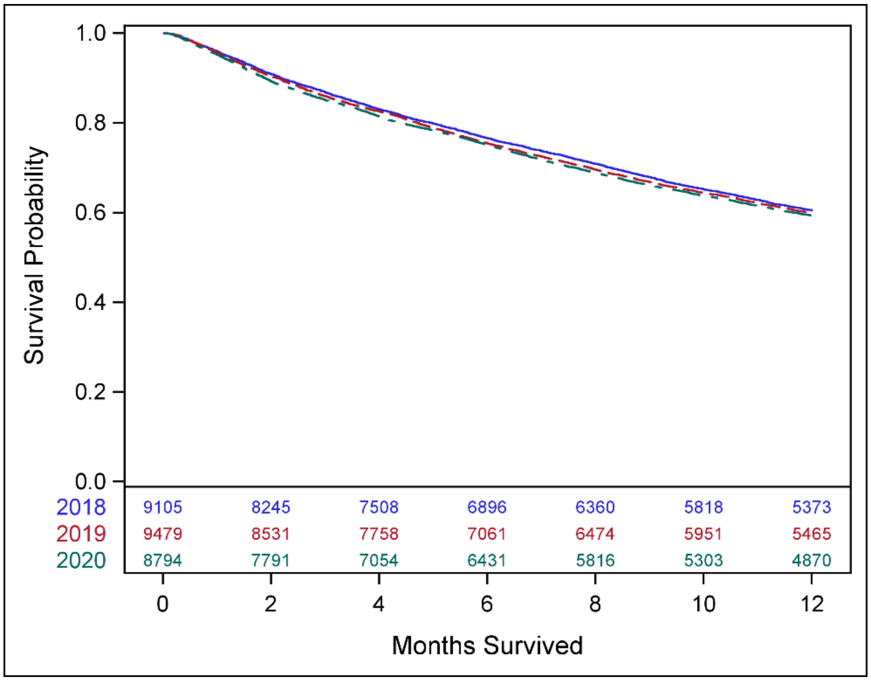

b) Gastric cancer

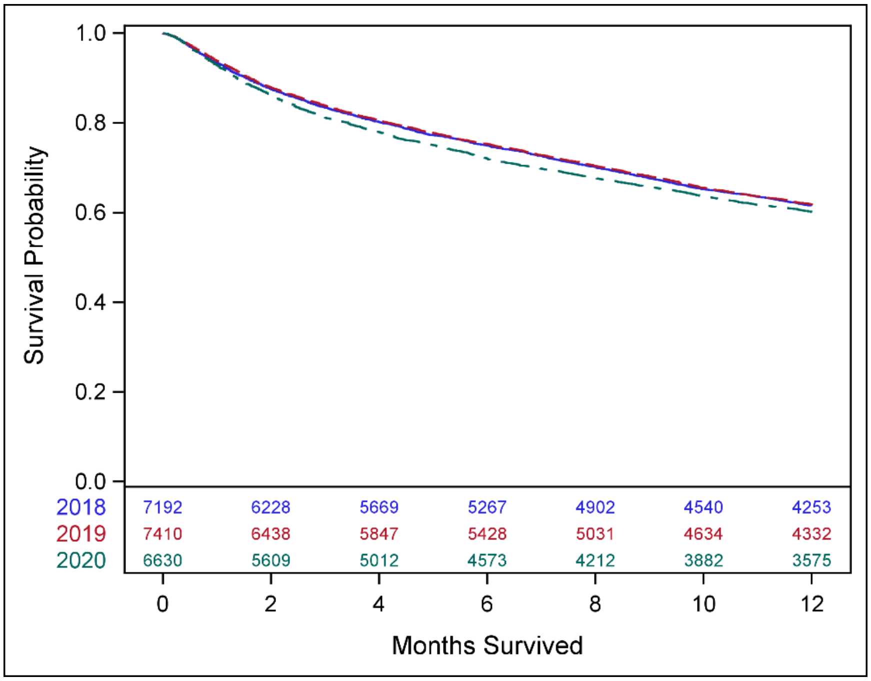

© 2024 Janczewski LM et al. *JAMA Network Open.*

c) Primary liver cancer

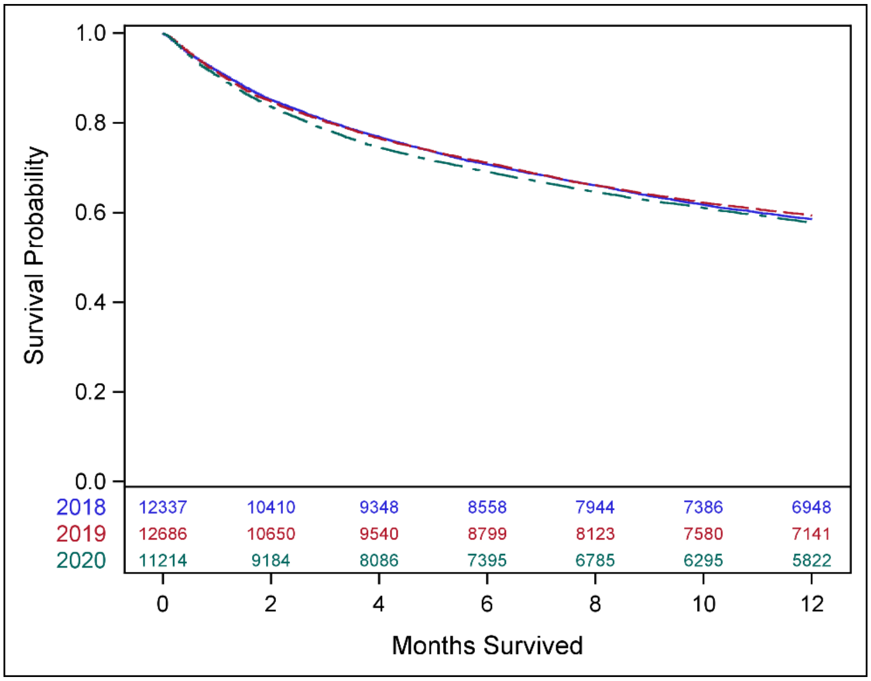

d) Pancreatic cancer

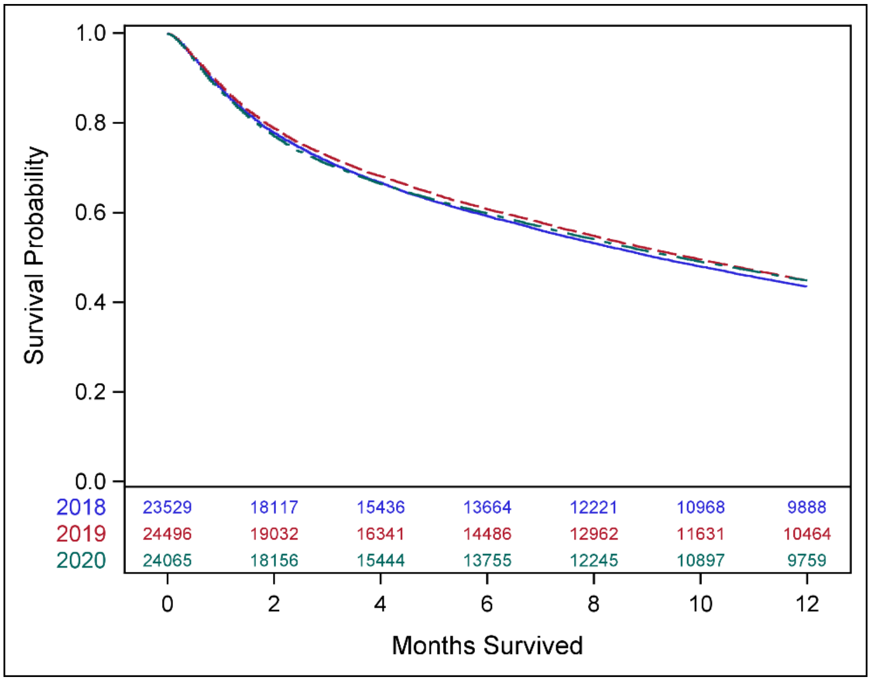

© 2024 Janczewski LM et al. *JAMA Network Open.*
