# Supplementary material for: Survival Among Patients With High-Risk Gastrointestinal Cancers During the COVID-19 Pandemic
Source: JAMA Netw Open. 2024 Mar 5;7(3):e240160. doi: 10.1001/jamanetworkopen.2024.0160 (PMC10915687; doi:10.1001/jamanetworkopen.2024.0160)
Supplement: Supplement 2. — Data Sharing Statement [file jamanetwopen-e240160-s002.pdf]

Data Sharing Statement

Janczewski. Survival Among Patients With High-Risk Gastrointestinal Cancers During the COVID-19 Pandemic. *JAMA Netw Open*. Published March 05, 2024.
doi:10.1001/jamanetworkopen.2024.0160

Data
Data available: No

Additional Information
Explanation for why data not available: The National Cancer Database is publicly available to Commission on Cancer facilities only as a part of accreditation.
